# A method for assessing carcinogenic risk of air fine particle-associated polycyclic aromatic hydrocarbons by considering bioaccessibility in lung fluids

**DOI:** 10.1016/j.mex.2019.03.009

**Published:** 2019-03-18

**Authors:** Xinlei Liu, Yuejiao Wang, Zelin Shen, Xuan Wu, Yu Shi, Fang Wang

**Affiliations:** a College of Environmental Science and Engineering, Ministry of Education Key Laboratory of Pollution Processes and Environmental Criteria, Tianjin Key Laboratory of Environmental Remediation and Pollution Control, Nankai University, Tianjin, 300350, China; b Tianjin Key Laboratory of Water Resources and Environment, Tianjin Normal University, Tianjin, 300387, China; c State Key Laboratory of Pollution Control and Resource Reuse, School of the Environment, Nanjing University, Nanjing, 210023, China

**Keywords:** Bioaccessibility-based approaches for air particle-associated PAHs assessment, Biochar fine particles, PM_2.5_, Polycyclic aromatic hydrocarbons, Simulated lung fluids, Bioaccessibility, Carcinogenic risk assessment

## Abstract

This study was conducted to evaluate the inhalation carcinogenic risk of PAHs in biochar fine particles using total concentration-based assessment approach and bioaccessibility-based assessment approach. Only limit PAHs in particles can be released in simulated lung fluids, leading to a low bioaccessibility (only ranging from 0.34% to 1.48% for biochar fine particles and from 3.21% to 44.2% for PM_2.5_), which would significantly affect health risk assessment. Therefore, bioaccessibility should always be favored over more traditional evaluations based on total concentration, while evaluating inhalation health risks of biochar-bound PAHs. To prove the broad applicability of bioaccessibility-based assessment approaches, we also compared health risk of actual atmospheric particles (PM_2.5_ collected from Nanjing, China) using total concentration-based approaches and bioaccessibility-based approaches.

• Proposed bioaccessibility-based approaches for assessing biochar risk are more accurate than traditional total concentration-based approaches;
• Proposed bioaccessibility-based approaches can be applied to health risk assessment of actual air particles;
• A more practical method was proposed to evaluate the bioaccessibility of PAHs in biochar fine particles or other specific component of atmospheric particle matters: using wet sieving method to prepare fine particles, using volatile organic solvent-drying method to load ^14^C-PAHs on fine particles, and using desorption experiments to determine bioaccessibility of PAHs.

## Graphical abstract

**Figure:**
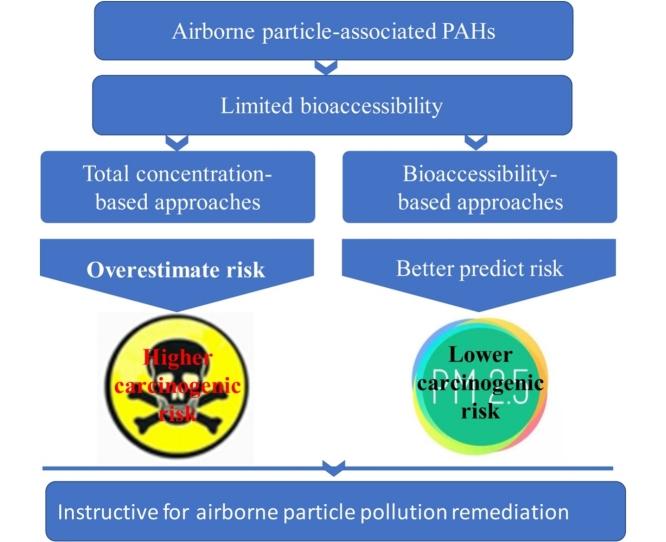

**Specifications Table**

**Table:** 

**Subject Area:**	Environmental Science
**More specific subject area:**	Health risks assessment
**Method name:**	Bioaccessibility-based approaches for air particle-associated PAHs assessment
**Name and reference of original method**:	Total concentration-based approaches for air particle-associated PAHs assessment

## Method details

### Background

Air fine particles are attracting increasing attention because of their potential impact on human health. The adverse health effects of air fine particles are thought to largely depend on particle-associated contaminants, such as polycyclic aromatic hydrocarbons (PAHs). When assessing exposure risks of contaminants associated with inhalation of air particles, the default assumption presumes that all contaminants in inhaled air particles are solubilized in lung fluid. However, contaminant bioaccessibility may be significantly reduced due to the binding of contaminants to air particles and their limited release under lung fluid conditions [1,2]. As a result, the influence of contaminant bioaccessibility in refining exposure and risks should be considered when assessing air particles. As one important component of atmospheric particulate matter [3], biochar can contain or accumulate an abundant of PAHs because of their high adsorption affinity [[4], [5], [6]]. To clarify how bioaccessibility affect the health risk of biochar-bound PAHs, daily intake (DI) method based on models recommended by the Environmental Protection Agency of United States (U.S. EPA) [7,8] was used to comparing cancer risk of PAHs by considering or not bioaccessibility in simulated lung fluids. Besides, to prove the broad applicability of bioaccessibility-based assessment approaches, we also compared cancer risk of actual atmospheric particles (PM_2.5_ collected from Nanjing, China) using total concentration-based approaches and bioaccessibility-based approaches.

## Materials and methods

### Assessing the bioaccessibility of particle-bound PAHs in simulated lung fluids

With respect to the inhalation route, a particle may reside in one of at least two “compartments”: the extracellular environment typified by lung fluid of neutral pH and the more acidic environment within macrophages [1]. Gamble’s solution and artificial lysosomal fluid (ALF) have been widely used for the exposure assessment of humans to inhalable pollutants, which can simulate different interstitial conditions in the lung. Gamble’s solution simulated extracellular lung fluid and ALF simulated intracellular lung fluid [9]. The initial formula has been modified by several researchers, such as simulated epithelial lung fluid (SELF), developed by Boisa et al. [1]. In this study, Gamble’s solution and ALF were used to measure inhalation bioaccessibility of PAHs in biochar fine particles, and SELF was used to measure inhalation bioaccessibility of PAHs in PM_2.5_. The bioaccessibility of PAHs in particles was calculated as following:

(1) $${\text{F}}_{\text{bioa}}\text{=}\frac{\text{PAH released in simulated lung fuid}}{\text{Total PAH in biochar fine particles}}$$

**Fig. 1:**
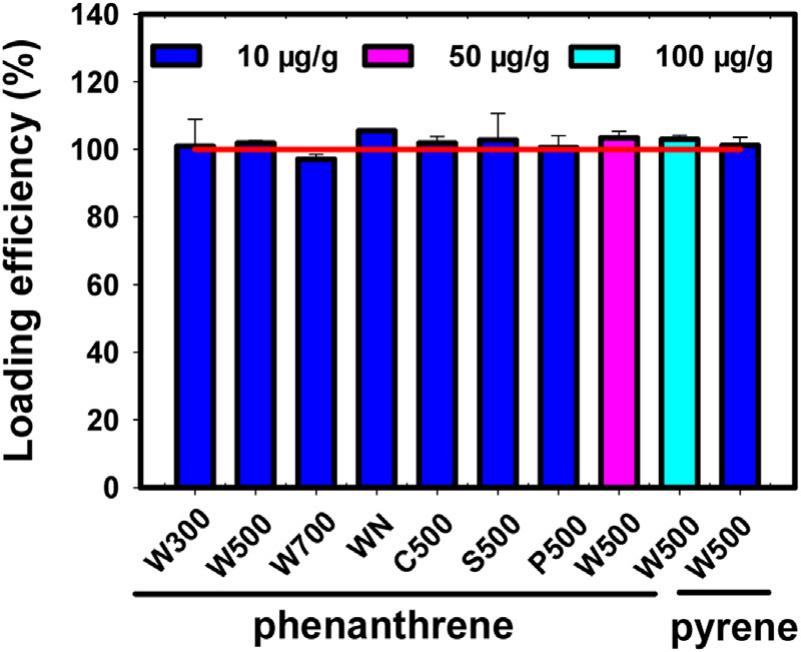
Loading efficiencies of PAHs on biochar fine particles.

Due to the limitation of current approaches for sample preparation and determination, there is few methods yet to accurately evaluate the bioaccessibility of contaminants in biochar fine particles. This study proposed an accurate and practical approach to evaluate bioaccessibility of biochar-bound PAHs: using wet sieving method to prepare biochar fine particles, using volatile organic solvent-drying method to load ^14^C-PAHs on biochar fine particles, and using desorption experiments to determine bioaccessibility of PAHs. Ten types of biochars were produced *via* pyrolyzing biomass at various HTTs under oxygen-limited conditions: W300, W500, W700 (wheat straw); C300, C500, C700 (corn straw); S300, S500, S700 (shaddock peel); P500 (peanut shell), wherein the numbers represent the pyrolysis temperature of the feedstocks in °C. Two types of biochars were produced by naturally burning wheat straw and corn straw, which is denoted as WN and CN. Phenanthrene and pyrene were selected as the model PAHs, because these two PAHs were reported to be two of the most abundant PAHs in biochars [4,6,10]. The PAH-loaded biochar fine particles were prepared using the volatile organic solvent-drying method, details of which have been previously reported [11]. The loading efficiencies of ^14^C-labled PAHs on the biochars were measured to verify that PAHs could be completely loaded on biochars (Fig. 1). This method is the most commonly used approach to prepare PAH-bearing carbon black, soot and other airborne fine particles, as reported in many studies on biological effects of PAH-bearing carbonaceous fine particles [[12], [13], [14], [15]]. Furthermore, the measured distribution coefficients (*K*_d_) for the desorption of phenanthrene and pyrene in electrolyte solutions are generally consistent with the values reported in the literature, conducted using biochar samples without PAH spiking [10,16], showing the feasibility of the loading method.

Data of PAHs in PM_2.5_ were from previous study of Li et al. [17]. PM_2.5_ samples were collected from Nanjing, China, and details of the sampling area, sampling technique, PAHs instrumental and data analysis have been previously reported [17]. Sixteen precedence-controlled PAHs set by U.S. EPA, *i.e.* naphthalene (Nap), acenaphthylene(Acl), acenaphthene (Ace), fluorene (Flu), phenanthrene(Phe), anthracene (Ant), fluoranthene (FA), pyrene (Pyr), benzo(a)anthracene (BaA), chrysene (Chr), benzo(b)fluoranthene (BbF), benzo(k)fluoranthene (BkF), benzo(a)pyrene (BaP), indeno(1,2,3-cd)pyrene (Ind), dibenz(a,h)anthracene (DBahA), and benzo(g,h,i)perylene (BghiP) were chosen as the model PAHs for assessment. For these 16 PAHs, the average concentrations which were obtained by determining 46 of PM_2.5_ samples and average bioaccessibility which were obtained by determining 14 PM_2.5_ samples were listed in Table 3, Table 4.

### Carcinogenic risk assessment of biochar fine particle-associated PAHs

The human-health risks of the two model PAHs bound to biochar fine particles were assessed by comparing the daily intake (DI) with the carcinogenic effect level as indicated by the acceptable daily intake (ADI) [18]. The DI value (ng/kg/day) can be calculated as [1]:

(2) $$\text{DI=(}{\text{q}}_{\text{PAH}}\times {\text{F}}_{\text{bioa}}\text{×TR×}{\text{C}}_{\text{biochar}}\times {\text{V}}_{\text{resp}}\text{)/BW}$$

where *q*_PAH_ is the mass fraction of a PAH in biochar fine particles, ng/g; *F*_bioa_ is the bioaccessible fraction (the total extent of desorption) of biochar-bound PAH in lung fluids; TR is the tracheobronchial retention, and a value of 75% was applied in this study [19]; *C*_biochar_ is the concentration of biochar fine particles (g/m^3^); *V*_resp_ is the inhalation rate (m^3^/day), and a value of 20 m^3^ air per day for adults was used [20]; BW (kg) is the body weight, and an average adult body weight of 60 kg was assumed [21]. The ADI value can be calculated as:

(3) $$\text{ADI=}\text{CR}/\left({\text{IPF}}_{\text{B(a)P}}\times {\text{TEF}}_{\text{PAH}}\right)$$

where CR is the acceptable range of carcinogenicity risk (10^–6^ to 10^−4^ as suggested by U.S. EPA [22] and 10^-6^ was here chosen for an individual carcinogen as suggested by the U.S. EPA [23]); IPF_B(a)P_ is the inhalation potency factor for benzo(a)pyrene (3.9 × 10^–3^ (ng/kg/day)^–1^ as reported by OEHHA [24]); TEF is the toxicity equivalent factor, and the TEF of PAHs are presented in Table 3. Using Eq. (3), the maximum ADI was calculated to be 0.26 ng/kg/day for phenanthrene and pyrene.

### Carcinogenic risk assessment of PAHs in actual atmospheric particles

Similar to biochar fine particles, the DI value (ng/kg/day) of PM_2.5_-bound PAH can be calculated as [1]:

(4) $$\text{DI=(}{\text{C}}_{\text{PAH}}\times {\text{F}}_{\text{bioa}}\text{×TR×}{\text{V}}_{\text{resp}}\text{)/BW}$$

where *C*_PAH_ is the concentration of a PAH in PM_2.5_ (ng/m^3^). Using Eq. (3), the maximum ADI of 16 precedence-controlled PAHs was calculated and presented in Table 3.

Besides daily intake (DI) model, BaP-TEQ model has also been widely used to evaluate human health risks of contaminants in atmospheric particles. It was carried out by calculating the carcinogenic equivalent concentrations (BaP-TEQ) relative to BaP, which were calculated by multiplying the toxic equivalent factors (TEF) by the concentration of PAH in samples [17,25]. The health risk of PAH can be assessed by comparing the total concentration (T*C*_PAH_) or bioaccessible concentration (B*C*_PAH_) with the acceptable concentration (A*C*_PAH_) of PAH, which can be calculated as:

(5) $${\text{A}\text{C}}_{\text{PAH}}\text{=}\frac{\text{ATEQ}}{\text{TEF}}\text{=}\frac{\text{CR}/\text{UR}}{\text{TEF}}$$

Where TEF is the toxic equivalent factors, and the TEF of 16 precedence-controlled PAHs set by U.S. EPA are presented in Table 3 [26]; UR is the unit risk of 8.7 × 10^–5^ (ng/m^3^)^–1^ for the lifetime 70 years PAH exposure, assuming one exposed to the average level of one unit BaP concentration (1 ng/m^3^) suggested by WHO [27,28]. ATEQ is the acceptable toxic equivalent quantity, which is calculated to be 0.0115 ng/m^3^. A*C*_PAH_ values of 16 PAHs were calculated using Eq. (4) and listed in Table 4.

## Method validation

### Bioaccessibility of particle-bound PAHs in simulated lung fluids

Biochar-bound PAHs was difficult to release in simulated lung fluids, leading to extremely low bioaccessibility (only ranging from 0.43% to 2.67% in Gamble’s solution and from 0.34% to 1.31% in ALF as shown in Table 1, Table 2). Physicochemical properties of biochars can influence bioaccessibility of PAHs in biochar fine particles [5,11,29]. For example, bioaccessibility of PAHs appeared to decrease with the increase of HTTs under which the biochars were prepared. The bioaccessibility of pyrene (PAH with more benzene rings) was lower than that of phenanthrene indicating that PAHs with more benzene rings may have relatively low bioaccessibility. Black carbon (BC), such as biochar was reported as one of the main reasons for the inhibition of PAHs mobilization following assessment in simulated body fluids due to its strong adsorption for PAHs [11,30]. Surely, the extremely lower bioaccessibility of PAHs would obviously affect the inhalation risk evaluation of biochar fine particles.

**Table 1:** Bioaccessibility (*F*_bioa_) and daily intake (DI) of phenanthrene and pyrene at a median loading concentration (10 μg/g) from different biochar fine particles.

	Gamble's solution				ALF			
	Phenanthrene		Pyrene		Phenanthrene		Pyrene	
Biochars	*F*_bioa_ (%)	DI (ng/kg/day)	*F*_bioa_ (%)	DI (ng/kg/day)	*F*_bioa_ (%)	DI (ng/kg/day)	*F*_bioa_ (%)	DI (ng/kg/day)
W300	0.7541	0.1056	0.7520	0.1053	0.6006	0.0841	0.5021	0.0703
W500	0.7472	0.1046	0.6233	0.0873	0.6110	0.0855	0.4658	0.0652
W700	0.6569	0.0920	0.4694	0.0657	0.5479	0.0767	0.3610	0.0505
WN	0.7495	0.1049	0.7422	0.1039	0.5224	0.0731	0.6739	0.0944
C300	1.4870	0.2082	1.1273	0.1578	1.3102	0.1834	1.0855	0.1520
C500	0.6995	0.0979	0.9940	0.1392	0.6110	0.0855	0.9911	0.1387
C700	0.4701	0.0658	0.5321	0.0745	0.3537	0.0495	0.5172	0.0724
CN	1.3340	0.1868	0.4299	0.0602	1.2051	0.1687	0.4065	0.0569
S300	0.9255	0.1296	0.7838	0.1097	0.7448	0.1043	0.5986	0.0838
S500	0.7123	0.0997	0.5997	0.0840	0.5394	0.0755	0.3769	0.0528
S700	0.5127	0.0718	0.4791	0.0671	0.3710	0.0519	0.3374	0.0472
P500	0.7111	0.0996	0.5270	0.0738	0.4961	0.0694	0.4816	0.0674

**Table 2:** Bioaccessibility (*F*_bioa_) in Gamble’s solution and daily intake (DI) of phenanthrene and pyrene at three different loading concentration from biochar fine particles.

Biochars	*q*_phe_ (μg/g)	Phenanthrene		Pyrene	
		*F*_bioa_ (%)	DI (ng/kg/day)	*F*_bioa_ (%)	DI (ng/kg/day)
W500	10	0.7472	0.1046	0.6233	0.0873
	50	2.6700	1.8690	1.1021	0.7714
	100	1.0969	1.5356	1.2169	1.7037
C500	10	0.6995	0.0979	0.7813	0.1094
	50	1.4405	1.0084	0.5516	0.3861
	100	1.3398	1.8757	0.9940	1.3916
S500	10	0.7123	0.0997	0.5997	0.0840
	50	2.2603	1.5822	0.7867	0.5507
	100	1.7200	2.4080	0.7007	0.9810

The mean inhalation bioaccessibility of 16 precedence-controlled PAH compounds in PM_2.5_ was assessed to be 3.21% (BcF) to 44.2% (Acl) using SELF (Table 1). Similar to biochar fine particles, the inhalation bioaccessibility can be influenced by physicochemical properties of the compound as well as the nature of sorptive phases within the particle matrix. Bioaccessibility of those high molecular weight PAHs was lower than other PAHs due to their higher hydrophobicity. Li et al. [17] observed a strong negative correlation between PAH inhalation bioaccessibility and ratio of elemental carbon (EC, representing BC) with total organic carbon, *i.e.*, EC / (EC + OC) (Pearson's r = 0.53; p = 0.05). As a result, black carbon (BC) plays an important role in reducing bioaccessibility of particle-bound PAHs. The reduced mobilization of PAHs in lung fluid, especially for those PAHs with higher molecular weight and those particle samples with higher content of BC showed a strong impact on health risk assessment of PAHs.

### Risk assessment of biochar fine particle-PAHs by considering or not bioaccessibility

The carcinogenic risks of PAHs in biochar fine particles were assessed by comparing the estimated DI with carcinogenic effect level (*i.e.* ADI). Thus, if the calculated DI value is below the ADI, then, the risk is acceptable; otherwise, the risk is unacceptable. Two methods can be used to calculate DI values, one assuming that all the biochar-bound PAH is bioaccessible (*i.e. F*_bioa_ = 100%), as assumed in conventional DI models, and the other based on the realistic bioaccessibility of PAH as measured. Assuming an extreme biochar pollution scenario wherein the concentration of biochar fine particles is as high as 5.6 mg/m^3^ in the air [3], the DI values of PAH based on total concentration were 14, 70 and 140 ng/kg/day when the loading concentration was 10, 50 and 100 μg/g, respectively. They were obviously higher than ADI (0.26 ng/kg/day). However, the DI values of PAH based on bioaccessible concentration in environmental loading concentration (10 μg/g) [5] were calculated to be between 0.047 to 0.21 ng/kg/day, which were lower than ADI (Table 1). Even in relatively higher loading concentration (50 and 100 μg/g), the DI values of PAH were ranged from 0.084 to 2.41 ng/kg/day (Table 2), just slightly higher than ADI. Thus, the above analysis suggests that biochar-bound PAHs will possibly pose low risks from the inhalation pathways, due to the strong sequestration of PAHs in the biochar fine particles. To put another way, currently adopted models may significantly overestimate the risks of biochar-bound PAHs.

### Risk assessment of PM_2.5_-associated PAHs by considering or not bioaccessibility

The establishment of air quality standard is usually based on toxicity data using total contaminant concentration, rather than considering bioaccessibility that influence contaminant exposure. However, DI values calculated by considering the bioaccessibility of PAH ranged from 0.0015 to 0.14 ng/kg/day, obviously higher than those DI values calculated based on total concentration of PAH (0.0075–1.52 ng/kg/day) (Table 3). Considering bioaccessibility or not affected the risk level of PM_2.5_. Phenanthrene and pyrene were assessed to be toxic if bioaccessibility were not considered. However, they turned out to be no toxic if taking bioaccessibility into account. Although FA, BaA, Chr, BbF + BkF, BaP, Ind, DBahA and BghiP showed cancer risk no matter taking or not bioaccessibility into account, DI values of these PAHs based on bioaccessibility were 5.11–47.0 times lower than that based on total concentration. Therefore, only analyzing the total concentration of PAHs in PM_2.5_ are not enough to accurately assess the health risk of PM_2.5_.

**Table 3:** The toxic equivalent factors (TEF), acceptable daily intake (ADI), concentration (*C*_PAH_), bioaccessibility (*F*_bioa_), daily intake (DI) and cancer risk level of 16 precedence-controlled PAHs set by U.S. EPA based on bioaccessibility and total concentration.

PAHs	TEF [26]	ADI (ng/kg/day)	*C*_PAH_ (ng/m^3^)	*F*_bioa_ (%)	Bioaccessibility based		Total concentration based	
					DI (ng/kg/day)	Cancer risk	DI (ng/kg/day)	Cancer risk
Nap	0.001	0.2564	0.13	–	–	–	0.0325	
Acl	0.001	0.2564	0.07	38.7	0.0068		0.0175	
Ace	0.001	0.2564	0.03	19.6	0.0015		0.0075	
Flu	0.001	0.2564	0.26	17.4	0.0113		0.0650	
Phe	0.001	0.2564	2.30	21.3	0.1224		0.5750	Risky
Ant	0.001	0.2564	0.88	18.7	0.0412		0.2200	
FA	0.01	0.0256	2.96	19.6	0.1449	Risky	0.7400	Risky
Pyr	0.001	0.2564	2.24	19.1	0.1072		0.5600	Risky
BaA	0.1	0.0026	4.61	2.77	0.0319	Risky	1.1525	Risky
Chr	0.01	0.0256	4.68	6.81	0.0797	Risky	1.1700	Risky
BbF + BkF	0.2	0.0013	4.73	3.62	0.0428	Risky	1.1825	Risky
BaP	1	0.0003	2.88	5.94	0.0428	Risky	0.7200	Risky
Ind	0.1	0.0026	4.88	2.13	0.0259	Risky	1.2200	Risky
DBahA	1	0.0003	0.80	4.68	0.0094	Risky	0.2000	Risky
BghiP	0.01	0.0256	6.06	2.34	0.0355	Risky	1.5150	Risky

Using BaP-TEQ assessment model, the cancer risk level of PM_2.5_-PAHs were assessed by comparing T*C*_PAH_ or B*C*_PAH_ with the A*C*_PAH_ (Table 4). The mean T*C*_PAH_ of 16 PAH compounds in PM_2.5_ ranged from 4.03 to 102 ng/m^3^. The corresponding B*C*_PAH_ in simulated epithelial lung fluid was assessed to be 0.01-0.58 ng/m^3^, only 3.21% to 44.2% of T*C*_PAH_. The FA, Chr, Ind and BghiP were evaluated to be risk using traditional assessment model. However, their cancer risk would be acceptable if assessed considering bioaccessibility. This result consisted with previous methods used in assessing biochar fine particles and actual air particles, suggesting the broad applicability of proposed approaches and the necessary of considering bioaccessibility of particle-bound PAHs when assessing the health risk of PM_2.5_.

**Table 4:** The acceptable concentration (A*C*_PAH_), bioaccessibility (*F*_bioa_) concentration (*C*_PAH_), bioaccessible concentration (B*C*_PAH_) and cancer risk level of 16 precedence-controlled PAHs set by U.S. EPA using BaP-TEQ method.

PAHs	A*C*_PAH_	*F*_bioa_ (%)	*C*_PAH_ (ng/m^3^)	B*C*_PAH_ (ng/m^3^)	Cancer risk	
					Bioaccessibility based	Total concentration based
Nap	11.49	–	0.13	–	–	
Acl	11.49	38.72	0.07	0.03		
Ace	11.49	19.58	0.03	0.01		
Flu	11.49	17.45	0.26	0.05		
Phe	11.49	21.28	2.30	0.49		
Ant	11.49	18.72	0.88	0.16		
FA	1.15	19.58	2.96	0.58		Risky
Pyr	11.49	19.15	2.24	0.43		
BaA	0.11	2.77	4.61	0.13	Risky	Risky
Chr	1.15	6.81	4.68	0.32		Risky
BbF + BkF	0.06	3.62	4.73	0.17	Risky	Risky
BaP	0.01	5.94	2.88	0.17	Risky	Risky
Ind	0.11	2.13	4.88	0.10		Risky
DBahA	0.01	4.68	0.80	0.04	Risky	Risky
BghiP	1.15	2.34	6.06	0.14		Risky

Several studies recently proposed that the risk-based management of PAHs should consider bioaccessibility [31,32]. Our results echo this sentiment, suggesting bioaccessibility should always be favored over more traditional evaluations, such as total concentration. If contaminated sites are evaluated for inhalation health risk using total concentrations, the potential risk is substantially higher and in many cases this conclusion may be inaccurate leading to possible expensive remediation efforts. As the number of sites with possible inhalation health risk continues to rise globally [[33], [34], [35]], it is highly needed to accurately characterize and ultimately prioritize contaminated sites by risk assessors. The proposed bioaccessibility-based assessment methods can make up the shortage of traditional methods based on total concentration.

## Additional information

Only limit particle-bound PAHs can be released in simulated lung fluids, leading to a low bioaccessibility (only ranging from 0.34% to 2.67% for biochar fine particles and from 3.21% to 44.2% for PM_2.5_). Therefore, health risks of air particles, especially carbonaceous particles, such as biochar fine particles were assessed to be obviously lower if the bioaccessibility was taken into consideration. The results of this study suggest that risk assessment and toxicity predictions of PAHs in air particles may be refined by using the bioaccessibility-based approaches that have been used for PAHs in soil and sediment. PAH inhalation bioaccessibility may be influenced by physicochemical properties of the compound as well as the nature of sorptive phases within the particle matrix. However, atmospheric particulate is chemically complicated and contains different content of components exhibiting high affinity for PAHs, such as black carbon. Therefore, further efforts should focus on the bioaccessibility assessment on air particles with wide properties. The proposed sample preparation, bioaccessibility determination and risk assessment approaches used for biochar fine particles can be also applied to other specific component, such as soot, mineral dust and microplastic. Furthermore, the bioaccessibility-based rather than total concentration-based assessment approaches should be preferred when assessing the health risk of particle-associated contaminants. The bioaccessibility in the current study was derived from *in vitro* tests. However, particles that came into human body would make contact with the interstitial fluid within the deep lung or were phagocytized by alveolar and macrophages which would not be the case in reality as assumed by the present study. Therefore, the significance of the risk assessment conducted herein was just to demonstrate the impact of bioaccessibility on the assessment outcome and validate the feasibility of assessment model, but not the outcome itself. The bioaccessible fraction derived from the *in vitro* method should be further validated by future *in vivo* studies.
